# Cigarette smoke exposure facilitates allergic sensitization in mice

**DOI:** 10.1186/1465-9921-7-49

**Published:** 2006-03-29

**Authors:** Katrien B Moerloose, Lander J Robays, Tania Maes, Guy G Brusselle, Kurt G Tournoy, Guy F Joos

**Affiliations:** 1 Department of Respiratory Diseases, Ghent University Hospital, Ghent, Belgium

## Abstract

**Background:**

Active and passive smoking are considered as risk factors for asthma development. The mechanisms involved are currently unexplained.

**Objective:**

The aim of this study was to determine if cigarette smoke exposure could facilitate primary allergic sensitization.

**Methods:**

BALB/c mice were exposed to aerosolized ovalbumin (OVA) combined with air or tobacco smoke (4 exposures/day) daily for three weeks. Serology, lung cytopathology, cytokine profiles in bronchoalveolar lavage fluid (BALF) and on mediastinal lymph node cultures as well as lung function tests were performed after the last exposure. The natural history and the immune memory of allergic sensitization were studied with *in vivo *recall experiments.

**Results:**

Exposure to OVA induced a small increase in OVA-specific serum IgE as compared with exposure to PBS (P < 0.05), while no inflammatory reaction was observed in the airways. Exposure to cigarette smoke did not induce IgE, but was characterized by a small but significant neutrophilic inflammatory reaction. Combining OVA with cigarette smoke not only induced a significant increase in OVA-specific IgE but also a distinct eosinophil and goblet cell enriched airway inflammation albeit that airway hyperresponsiveness was not evidenced. FACS analysis showed in these mice increases in dendritic cells (DC) and CD4^+ ^T-lymphocytes along with a marked increase in IL-5 measured in the supernatant of lymph node cell cultures. Immune memory experiments evidenced the transient nature of these phenomena.

**Conclusion:**

In this study we show that mainstream cigarette smoke temporary disrupts the normal lung homeostatic tolerance to innocuous inhaled allergens, thereby inducing primary allergic sensitization. This is characterized not only by the development of persistent IgE, but also by the emergence of an eosinophil rich pulmonary inflammatory reaction.

## Background

Cigarette smoke can trigger acute symptoms in patients with asthma, and exposure to cigarette smoke is strongly correlated with asthma severity [1-3]. Animal models support these findings [4,5]. Recent evidence suggests that active smoking is a risk factor for the onset of adult asthma [6], but whether there is a causal relationship remains a matter of debate.

Although animal models are widely applied for the study of the immunopathology underlying asthma, the majority of them are based on artificial triggers to induce airway disease. It is well documented that the normal immune response to inhaled harmless allergens, both in mice and men, is tolerance [7,8]. Tolerance is a phenomenon that is mediated by active immune mechanisms [9]. To create a model of allergen induced airway inflammation, the normal tolerance thus has to be overcome. Mostly, an adjuvant such as aluminium hydroxide is therefore applied [10]. However, the clinical relevance of this approach is limited.

The question thus arises whether environmental noxious agents such as cigarette smoke could be one of the mechanisms responsible for the suppression of the tolerogenic status in asthma. Very few experimental data are available on this issue. One study in rats suggested that environmental tobacco smoke (ETS) could augment IgE responses to harmless allergens [11], while one other in mice showed that particles from sidestream cigarette smoke not only increased IgE, but also induced airway eosinophilia upon restimulation with allergen later on [12]. In contrast, Bowles *et al*. [13] indicated that ETS exposure accompanied by nose-only allergen exposure failed to overcome aerosol tolerance in three different mouse strains.

The objective of this study was therefore to determine whether mainstream cigarette smoke could inhibit inhalational tolerance and thus facilitate sensitization to a harmless antigen. We developed and characterized a new model in which mice were simultaneously exposed to aerosolized OVA and mainstream smoke, without any prior (aluminium-assisted) immunization.

## Methods

### Animals

Male inbred BALB/c mice of about eight weeks old were obtained from Harlan CBP (Zeist, the Netherlands). Food and water were provided ad libitum and mice were kept in a 12 h-light, 12 h-dark cycle. The local Ethical Committee (ECP Ghent University) approved the in vivo manipulations used in this study.

### In vivo tobacco smoke and allergen exposure

Mainstream cigarette smoke exposures were performed in a plexiglas chamber 17 cm × 28 cm × 14 cm with an inlet for pressurized air (1.25 L/min), connected to a smoking machine designed by Shapiro (St. Louis, MO) [14]. Groups of 8 mice were exposed to mainstream smoke of 5 Kentucky Reference cigarettes (2R4F without filter)(University of Kentucky, Lexington, KY, USA) for 7 min, 4 times a day. Carboxyhemoglobin-levels in blood were 8.29 ± 1.4% in smoke-exposed vs. 1.02 ± 0.22% in air-exposed mice (n = 7). During air or smoke exposure, concurrent challenges with phosphate buffered saline (PBS) or OVA (Grade III; Sigma Chemical Co., Poole, UK)(1% in PBS) were performed using an ultrasonic aerosol generator for 7 min, 4 times a day (Sirius Nova, Heyer Medizintechnologie, Bad Ems, Germany).

### Experimental protocols

#### 1) Acute exposure model

Groups of 8 mice received exposures as detailed above to either PBS and air, OVA and air, PBS and smoke or OVA and smoke. The exposures took place 5 days a week for three consecutive weeks. 24 hours after the last exposure, the mice were sacrified, samples were taken and lymph node cells were cultured.

#### 2) *In vivo *recall challenge with OVA

To test the immune memory responses 3 weeks after the last exposure, mice were re-exposed to 1% OVA aerosol for 30 min on 3 consecutive days. 24 hours after the last recall, the mice were sacrified for analysis.

### Bronchoalveolar lavage

Bronchoalveolar lavage (BAL) was performed via 3 intratracheal instillations with 0.3 ml Hank's balanced salt solution (HBSS; Pasteur, Brussels, Belgium) + 1% Bovine Serum Albumin, followed by 3 instillations with 1 ml of HBSS to collect cells for cytospin analysis. After staining with May-Grünwald-Giemsa, total and differential cell counts were done using standard morphologic criteria. BAL cells and cells from lung digest were analyzed by Fluorescence Activated Cell Sorter (FACS).

### Measurement of total and ovalbumin-specific IgE and IgG1

Blood was collected by cardiac puncture for measurement of total and OVA-specific IgE and IgG1 with ELISA as described before [5].

### Mediastinal lymph node cell culture

Paratracheal and parathymic intrathoracic lymph nodes were collected into sterile tubes containing cold (4°C) tissue culture medium (TCM) and digested to obtain a single cell suspension. These cells were then cultured in TCM in a flat-bottom, 96-well plate (Becton Dickinson (BD), CA, USA) alone or with OVA (0, 10 or 100 μg/ml), at a density of 8*10^5 ^cells per well. After 5 days, supernatants were harvested and frozen for cytokine measurements.

### Immunofluorescent labeling and flow cytometry

Lung digest was performed as described before [15] and cells were labeled for flow cytometry. In brief, cells of lavage fluid and the right lung were pre-incubated with Fc-receptor blocking antibody (anti-CD16/CD 32, clone 2.4G2) to reduce non-specific binding. Dendritic cells were identified as described before [16,17]. For identification of CD11c+ dendritic cells, biotinylated anti-CD11c (N418) was used, and for MHCII, anti-IA/IE (M5/114) was used.

Monoclonal antibodies used to identify lymphocytes were: biotinylated anti-CD3 (145-2C11), FITC-conjugated anti-CD4 (GK1.5), FITC-conjugated anti-CD8 (53–6.7), and PE-conjugated anti-CD69 (H1.2F3). Biotinylated antibodies were revealed using streptavidin-APC (Sav-APC). Before analysis, cells were incubated with 7-amino-actinomycin (7AAD or Viaprobe) 10 min at room temperature for dead cell exclusion. All reagents were obtained from BD Pharmingen (Erembodegem, Belgium). Flow cytometry data were acquired on a FACSVantage SE flow cytometer running CELLQuest 3.0™ (BD). FlowJo software (Treestar, CA, USA) was used for data analysis.

### Cytokine and chemokine measurements

On supernatant of lavage fluid, IL-13, tumor necrosis factor-α (TNF-α), granulocyte-macrophage colony-stimulating factor (GM-CSF), thymus- and activation regulated cytokine (TARC/CCL17) and IFN-γ were measured with ELISA (R & D, Abingdon, UK). Cytometric Bead Array (CBA, BD) was used to detect the cytokines interleukin (IL)-4, IL-5, IL-10 and IL-13 in supernatants of lymph node cell cultures.

### Histologic analysis

Formalin-fixed, paraffin-embedded lung lobes were cut into 3 μm sections and stained with Congo red or periodic acid-Schiff (PAS) to highlight eosinophils and goblet cells, respectively. Quantification of inflammation was performed in a blinded fashion using a Zeiss KS400 Image Analyzer (Oberkochen, Germany) running a custom-made morphometry program. For each mouse, digital images of five bronchi were acquired. Quantitative measurements were performed in all airways with a perimeter of basement membrane (Pbm) between 800 and 2000 μm and cut in reasonable cross sections (ratio of maximal to minimal internal diameter smaller than 1.8). Morphometrical parameters, like the perimeter of the basement membrane (Pbm) were marked manually on the digital representation of the airway, as described before [18,19].

### Assessment of airway responsiveness

Twenty-four hours after the final exposure, lung resistance at baseline and that induced by increasing doses of carbachol, was evaluated with a computerized pulmonary mechanics analyzer (Mumed lung function recording system, version 5.0, 1999, Mumed systems Ltd., London, U.K.) as described before [5]. Carbachol-induced bronchoconstriction was measured as the percentage increase in lung resistance (RL), comparing the peak of the reaction with baseline RL.

### Statistical analysis

Each group contained 8 mice. Reported values were expressed as mean ± standard error of the mean (SEM). P-values of less than 0.05 were regarded as significant. For the statistical analysis, SPSS 11.0 was used (SPSS Inc. Chicago, IL, USA). All outcome variables were compared using non-parametrical tests (Kruskal-Wallis; Mann-Whitney U-test with Bonferroni's corrections).

## Results

### Total and OVA-specific IgE and IgG1 in serum

**Figure 1:**
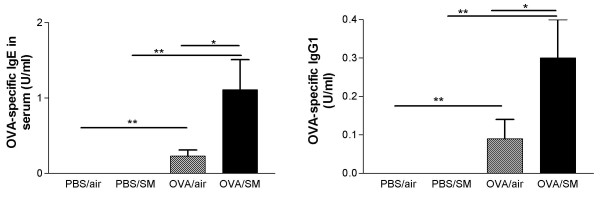
Serum ovalbumin-specific IgE and IgG1 (U/ml) in mice exposed to phosphate buffered saline (PBS) or ovalbumin (OVA) combined with air or cigarette smoke (SM) for three weeks. (n = 8 per group. Values are reported as mean ± SEM) (*P < 0.05; **P < 0.001).

Total IgE in serum was not increased in mice exposed to either OVA or cigarette smoke as compared to PBS/air-exposed mice (0.07 ± 0.02 μg/ml). In mice exposed to both OVA and cigarette smoke, a small increase in total IgE (0.14 ± 0.01 μg/ml) was observed vs. smoke-exposed (0.07 ± 0.01 μg/ml; P < 0.05), but not vs. OVA-exposed mice (0.10 ± 0.02 μg/ml). OVA-specific IgE and IgG1 was significantly higher in mice exposed to OVA vs. PBS. Simultaneous exposure to OVA and cigarette smoke caused a further increase in the OVA-specific IgE (Figure 1A) and IgG1 (Figure 1B) (P < 0.05).

### Bronchoalveolar lavage fluid

**Figure 2:**
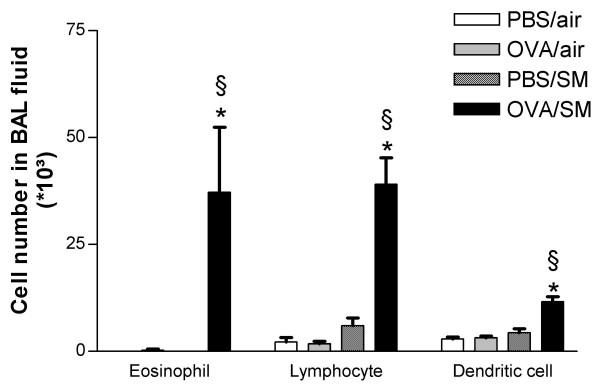
Number of eosinophils, lymphocytes and dendritic cells in bronchoalveolar lavage fluid of mice exposed to phosphate buffered saline (PBS) or ovalbumin (OVA) combined with air or cigarette smoke (SM) for three weeks. (n = 8 per group. Values are reported as mean ± SEM) (*P < 0.01 vs. OVA/air-exposed group; §P < 0.01 vs. PBS/smoke-exposed group).

Neither OVA nor smoke exposure during three weeks caused a significant change in total BAL cell number, as compared to PBS- and air-exposed animals (542.5 × 10^3 ^± 64.5 × 10^3 ^and 805.0 × 10^3 ^± 122.1 × 10^3 ^vs. 607.5 × 10^3 ^± 74.2 × 10^3 ^respectively; P > 0.05). However, in mice simultaneously exposed to OVA and cigarette smoke, an increased total cell number was seen in the BAL fluid (1090.0 × 10^3 ^± 92.0 × 10^3^; P < 0.001 vs. OVA/air-exposed mice). Smoke exposure as such increased the number of neutrophils in BAL fluid (3.8 × 10^3 ^± 0.5 × 10^3 ^vs. 0.6 × 10^3 ^± 0.3 × 10^3 ^in air-exposed mice; P < 0.01)(12.8 × 10^3 ^± 5.1 × 10^3 ^in OVA- and smoke-exposed mice vs. 0.9 × 10^3 ^± 0.4 × 10^3 ^in OVA- and air-exposed mice; P < 0.05). Exposure to either OVA or cigarette smoke alone did not induce eosinophil, lymphocyte and DC influx in BALF as compared to PBS exposure (Figure 2). The combination of cigarette smoke and OVA caused a significant increase in the number of these three cell types recovered from BAL (P < 0.01 compared to all treatments) (Figure 2).

OVA or smoke exposure as such did not affect the number of macrophages in BAL fluid, but mice exposed simultaneously to OVA and smoke showed higher BAL macrophage numbers compared to PBS/air-exposed mice (1000.3 × 10^3 ^± 91.6 × 10^3 ^vs. 604.6 × 10^3 ^± 74.3 × 10^3^; P < 0.01) and to OVA/air-exposed mice (539.4 × 10^3 ^± 64.7 × 10^3^; P < 0.01), but not compared to PBS/smoke-exposed mice (795.0 × 10^3 ^± 120.5 × 10^3^).

### Cellular changes in lung tissue

**Figure 3:**
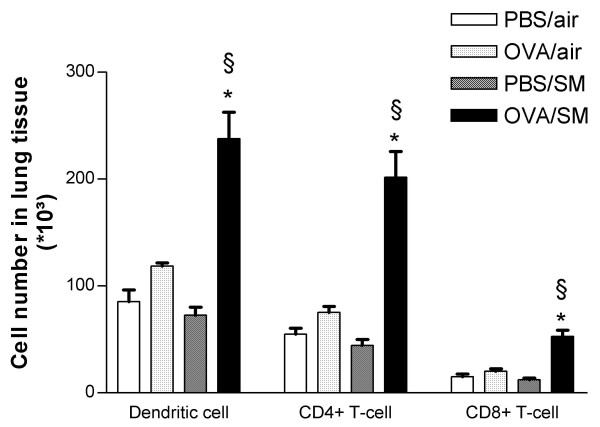
Number of dendritic cells, CD4+ T-lymphocytes and CD8+ T-lymphocytes in lung tissue of mice exposed to phosphate buffered saline (PBS) or ovalbumin (OVA) combined with air or cigarette smoke (SM) for three weeks. (n = 8 per group. Values are reported as mean ± SEM) (*P < 0.01 vs. OVA/air-exposed group; §P < 0.01 vs. PBS/smoke-exposed group).

**Figure 4:**
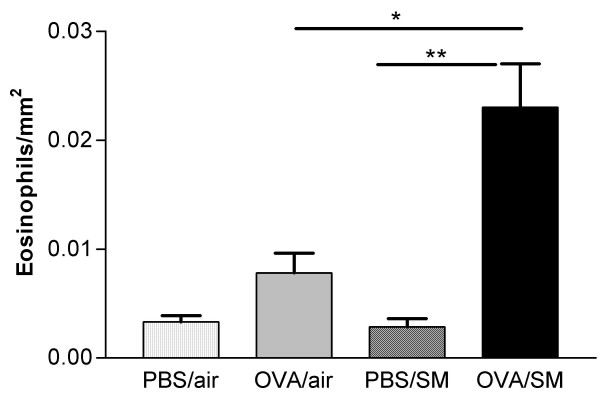
Eosinophils per mm^2 ^in the airway wall of mice exposed to phosphate buffered saline (PBS) or ovalbumin (OVA) combined with air or cigarette smoke (SM) for three weeks. (n = 8 per group. Values are reported as mean ± SEM) (*P < 0.01; **P < 0.001).

**Figure 5:**
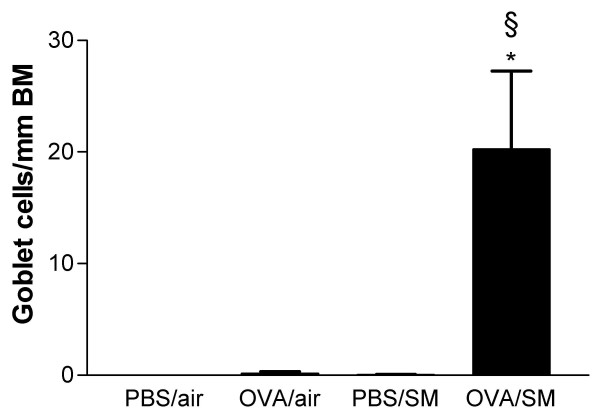
Goblet cells in the airway wall of mice exposed to phosphate buffered saline (PBS) or ovalbumin (OVA) combined with air or cigarette smoke (SM) for three weeks. Definition of abbreviations: Pbm = perimeter of basement membrane (n = 8 per group. Values are reported as mean ± SEM) (*P < 0.01 versus OVA/air-exposed group; §P < 0.001 vs. PBS/smoke-exposed group).

**Figure 6:**
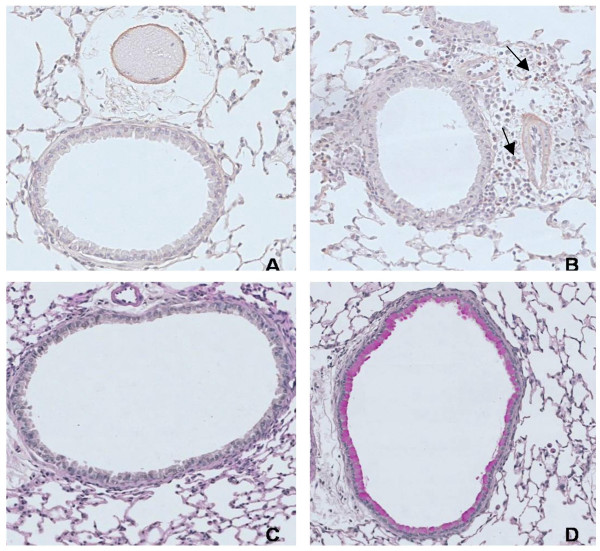
Eosinophils and goblet cells in the airway wall of mice exposed to phosphate buffered saline (PBS) or ovalbumin (OVA) combined with air or cigarette smoke (SM) for three weeks. A. Congo red staining for eosinophils in OVA- and air-exposed mice. B. Congo red staining for eosinophils in simultaneously OVA- and smoke-exposed mice. The arrows are indicating eosinophils. C. Periodic acid-Schiff staining for goblet cells in OVA- and air-exposed mice. D. Periodic acid-Schiff staining for goblet cells in simultaneously OVA- and smoke-exposed mice.

In mice exposed to both OVA and cigarette smoke, the number of dendritic cells, activated CD4+ and activated CD8+ T-lymphocytes in lung tissue were more than doubled compared to all other groups (Figure 3). Histologic analysis showed that mice exposed to OVA and cigarette smoke developed peribronchial infiltrates that contained increased numbers of eosinophils as compared to all other groups (Figure 4; Figure 6A and 6B). On PAS-stained tissue sections, neither OVA nor smoke exposure as such induced goblet cell hyperplasia in BALB/c mice. In mice that were simultaneously exposed to both stimuli, a massive goblet cell hyperplasia was observed (20.26 ± 7.7 goblet cells/μm basement membrane)(Figure 5; Figure 6C and 6D).

### Cytokine and chemokine levels in BALF supernatant

**Figure 7:**
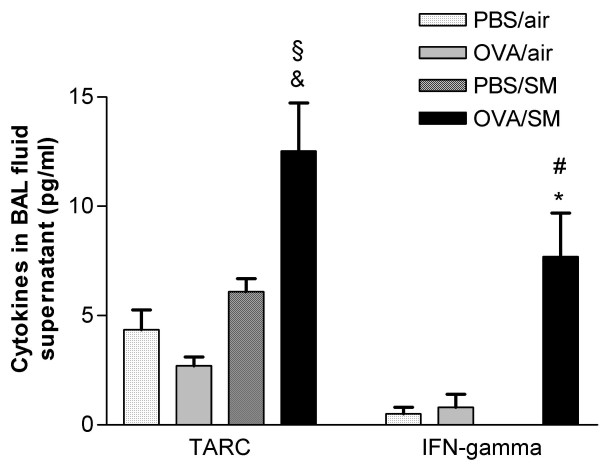
Cytokines in BAL fluid supernatant (pg/ml) of mice exposed to phosphate buffered saline (PBS) or ovalbumin (OVA) combined with air or cigarette smoke (SM) for three weeks. Definition of abbreviations: TARC = thymus and activation regulated chemokine (n = 8 per group. Values are reported as mean ± SEM) (*P < 0.01, & P < 0.001 vs. OVA/air-exposed group; §P < 0.01, #P < 0.001 vs. PBS/smoke-exposed group).

TARC/CCL17 and IFN-γ levels in BALF did not increase significantly in the groups exposed to either OVA or cigarette smoke as compared to PBS- and air-exposed mice. However, simultaneous exposure to OVA and smoke significantly augmented TARC and IFN-γ levels in BALF as compared to all other groups (Figure 7). There were no significant differences in GM-CSF (1.95 ± 0.84 vs. 1.87 ± 1.08 pg/ml) between the mice exposed to OVA and smoke versus OVA as such. IL-13 and TNF-α were below detection limit in all groups.

### Cytokine levels in supernatants of lymph node cell cultures

**Figure 8:**
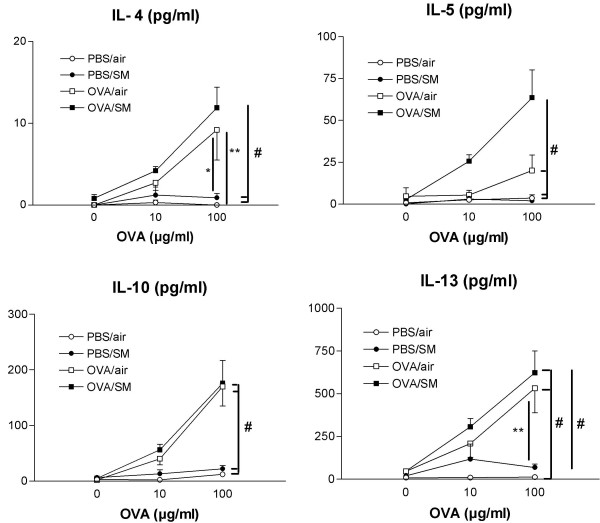
Cytokines IL-4, IL-5, IL-10 and IL-13 in lymph node cell culture supernatant (pg/ml) after stimulation with OVA (0, 10 and 100 μg/ml). Mice were exposed to phosphate buffered saline (PBS) or ovalbumin (OVA) combined with air or cigarette smoke (SM) for three weeks. (n = 8 per group. Values are reported as mean ± SEM) (* P < 0.05, ** P < 0.01,# P < 0.001).

Since a distinct allergic inflammation was observed in mice concurrently exposed to OVA and smoke, we investigated cytokines in the mediastinal lymph nodes. Therefore, lymph node cell cultures were stimulated with 0, 10 or 100 μg/ml OVA, and a dose-dependent increase in IL-4, IL-5, IL-10 and IL-13 was observed in all mice that were at first challenged with OVA, regardless of the presence of cigarette smoke (Figure 8). IL-5 was significantly increased only in the mice exposed to both OVA and smoke (Figure 8).

### Airway responsiveness

In the acute experiment, no significant differences were observed between the four groups in baseline lung resistance (RL = 359.35 ± 9.58 cm H2O/liter/sec = mean of baseline resistances of the four groups). After three weeks of OVA and/or smoke exposure, the dose-response curve for carbachol was not significantly different from those of naïve animals (data not shown).

### In vivo recall challenge with ovalbumin

To investigate whether the adjuvant-like effects of cigarette smoking were long-lasting, an *in vivo *rechallenge experiment was performed. 4 groups of 8 mice were subjected to the same exposures as in the acute experiment, and after a resting period of 3 weeks, all mice were exposed to 1% OVA aerosol for 30 min on 3 consecutive days.

After the in vivo recall challenges, OVA-specific IgE was higher in mice that were at first exposed to both OVA and cigarette smoke (1.28 ± 0.4 U/ml in OVA/smoke- vs. 0.00 ± 0.0 in PBS/air- and in PBS/smoke-exposed mice; P < 0.05, and vs. 0.20 ± 0.07 in OVA/air-exposed mice; P < 0.05).

The cell numbers and differential cell counts in BALF were comparable between the four groups and compared also with the numbers in the negative control group (PBS/air) of the acute experiment (Table 1). No significant peribronchial infiltrates of eosinophils were reported in any of the four groups (data not shown), the inflammatory reaction of the OVA/smoke group had disappeared completely. Only the number of goblet cells was increased in the OVA- and smoke-exposed mice after the recall (2.62 ± 0.8 goblet cells/μm basement membrane in OVA/smoke vs. 0.60 ± 0.3 in PBS/air; P < 0.05, vs. 0.23 ± 0.1 in PBS/smoke; P < 0.05 and vs. 0.33 ± 0.2 in OVA/air; P < 0.05), but this phenomenon was almost 10-fold less than after the acute exposures.

**Table 1:** Total cell number, number of eosinophils, lymphocytes, macrophages and neutrophils in bronchoalveolar lavage fluid of mice exposed to phosphate buffered saline (PBS) or ovalbumin (OVA) combined with air or cigarette smoke (SM) for three weeks. Three weeks after the last exposure, all mice were rechallenged in vivo to 1% OVA aerosol on 3 consecutive days. (n = 8 per group. Values are reported as mean ± SEM. No significant differences were observed.)

	PBS/air	PBS/smoke	OVA/air	OVA/smoke
Total cell number (*10^3^)	445.7 ± 41.0	475.0 ± 49.8	525.0 ± 25.5	470.0 ± 35.4
Eosinophils	0.0 ± 0.0	0.0 ± 0.0	0.0 ± 0.0	1065.0 ± 968.8
Lymphocytes	367.7 ± 186.6	168.0 ± 168.0	2507.5 ± 825.1	5448.0 ± 2932.2
Macrophages (*10^3^)	444.3 ± 41.0	473.5 ± 49.8	520.3 ± 25.1	460.5 ± 36.0
Neutrophils	964.0 ± 432.1	1322.7 ± 281.6	2113.5 ± 848.0	2622.5 ± 1597.9

## Discussion

In this study, we provide further evidence for the hypothesis suggesting that exposure to cigarette smoke can disrupt the normal tolerogenic immune responses against harmless antigens. Our data suggest that cigarette smoke can be depicted not only as an aggravating factor for existing airway inflammation [5], but also as a facilitating and evitable factor in the development of allergic airway disease.

Repeated exposure to aerosolized harmless inert antigens such as OVA or pollen allergens without a prior systemic, adjuvant aided sensitization does in naïve mice not lead to pulmonary disease, although small increases in IgE have been noted [7,12,20,21]. The current results underscore these findings with the documentation of low serum OVA-specific IgE values but no airway disease upon OVA exposure. This means that, in the absence of additional signals, endogenous tolerogenic mechanisms hold control [9].

These additional signals may vary in nature. Some allergens contain intrinsic enzymatic activity (e.g. protease activity in house dust mite extracts), which can raise endogenous signals while other additional signals function independent of allergen (e.g. respiratory viruses). However, they are all potentially responsible for the suppression of normal tolerance [22]. In experimental models, the adjuvant aluminium hydroxide is also known to overcome the normal inhalational tolerance against inert proteins such as OVA or pollen allergens.

Cigarette smoking represents a real social problem with enormous health consequences. Exposure to tobacco smoke causes chronic obstructive pulmonary disease and lung cancer. Furthermore, it increases the severity of asthma but its role in asthma development is more controversial. Along with mouse models of COPD and emphysema [17], it has now become feasible to evaluate the potential role of cigarette smoke as clinically relevant danger signal in the natural history of asthma. Short-term exposure to cigarette smoke (3 weeks) does in our hands not lead to overt pulmonary disease or emphysema, the latter being evidenced after long term exposure [17]. Nevertheless, we observed a small but significant increase in the number of neutrophils in BALF suggestive for an acute toxic effect.

We thus combined this cigarette smoke exposure protocol with OVA aerosols in naive animals and show that besides a significant additive effect on the development of specific IgE and IgG1, also active and eosinophil enriched pulmonary inflammation develops. Others also used naive mice to demonstrate that exposure to aerosolized cigarette smoke extracts could induce an augmentation in IgE to an otherwise innocuous antigen [12]. In the latter report, smoke extracts were generated from sidestream smoke and mixed with saline for delivery through a nebulizer. This differs from our study where pure cigarette fumes are administered directly and concomitant with allergens. However, both studies underscore the fact that cigarette smoke effectively can act as an adjuvant to facilitate primary allergic sensitization to inert antigens. A possible mechanism for the induction of sensitization by mainstream smoke is that cigarette smoke extracts improve the antigen presentation either by adsorption of allergen, thereby causing a more persistent allergen exposure, or by causing structural modifications to the allergen itself. These mechanisms have also been suggested for the adjuvant effects observed with carbon black and diesel particles [23]. In addition, cigarette smoke particles may have an irritative effect on the epithelial surface, causing damage to the mucosae and facilitating the penetration of allergen into the epithelial layer. A contiguous network of dendritic cells (DC) is present underneath the respiratory epithelium ready to take up and process antigen, and to migrate to the draining lymph nodes for presentation to antigen specific T-cells for triggering an active immune response [16]. The involvement of DC in the observed pathology is suggested by the significant increase of DC in both BALF and lung tissue in the OVA- and smoke-exposed mice. These DC are responsible for priming and activation of CD4^+ ^T-cells in bronchial lymph nodes, which recirculate via the bloodstream towards the lungs where we could measure them (BALF and lung tissue). Not only increases in DC and T-cells, but also a substantial amount of eosinophils and to a far lesser extent of neutrophils was noticed only in the mice exposed to the combination of cigarette smoke and allergen. In addition, we found a remarkable presence of goblet cells in these airways. In this model, we were not able to demonstrate airway hyperresponsiveness. Although methodology of pulmonary function assessment might be important, another possible explanation is that the time point of testing was not optimal. On the other hand, it could be that there was simply no hyperresponsiveness, possibly because the strength of the inflammatory reaction was insufficient. This is plausible since the magnitude of the inflammatory response both in terms of serum IgE and pulmonary inflammation is much less as compared to the inflammatory responses observed after systemic priming (with aluminium hydroxide) and airway exposures.

At the molecular level, we found that TARC was elevated in BALF supernatant of the mice simultaneously exposed to allergen and cigarette smoke, whereas it remained stable after exposure to either ovalbumin or smoke alone. Kawasaki *et al*. [24] reported that TARC is a pivotal chemokine for the development of Th2-dominated experimental allergen-induced asthma with eosinophilia, which fits with our findings. Besides TARC, GM-CSF also mediates, at least in part, Th2 sensitization and eosinophilic airway inflammation [20,25]. We were unable to detect differences in BALF GM-CSF which contrasts with the findings of Rumold *et al*. [12], who suggested this cytokine as well as IL-5 as a possible explanation for eosinophilia observed after a rechallenge with allergen. Intruigingly, the Th-1 cytokine IFN-γ was increased in BALF of OVA/smoke-exposed mice. Although this most probably reflects the toxic effects of cigarette smoke and parallels with the neutrophilic inflammatory reaction, it is to note that IFN-γ did not abolish the eosinophil rich inflammation as could be expected in the first instance [26]. In addition, elevated levels of IFN-γ were reported in serum [27], BAL fluid [28] and in sputum [29] of patients with asthma, all suggesting that some of the pathology in asthma could partially be IFN-γ driven.

The Th2 cytokines IL-4, IL-10 and IL-13 (not detectable in BALF) were augmented in lymph node cell culture supernatant in all mice that were initially exposed to ovalbumin. This suggests that, similar to what was already described for OVA-specific IgE, a transitory rise in these T-cell produced cytokines occurs at the level of the lymph nodes, which is however not strong enough to induce pulmonary disease. IL-5, another predominant Th2 cytokine, was upregulated only in those mice exposed to both OVA and cigarette smoke. This finding explains the eosinophilia in the BALF and lung tissue and again closes up the hypothesis that DC stimulate allergen specific T-cells to produce IL-5 upon exposure to allergen and cigarette smoke.

Whether the observed breakdown of immunological homeostasis after concomitant allergen and cigarette smoke exposure with the ensuing pulmonary eosinophil rich inflammation has a memory character was investigated by performing *in vivo *recall experiments. Re-challenge of the mice with allergen alone after a resting period of 3 weeks showed that the normal tolerogenic mechanisms were restored, with a complete absence of pulmonary inflammation. Several explanations are possible for this observation: firstly, the sensitization to OVA facilitated by cigarette smoke was not long lasting. In this case the OVA-specific IgE and the goblet cell hyperplasia was a remainder of the acute inflammatory reaction. Another possibility is that the rechallenge period was too short to re-induce a marked airway inflammation. Further investigation will have to shed a light on these findings.

In conclusion, in an acute mouse model we have shown for the first time that mainstream cigarette smoke directly disrupts the normal lung homeostatic tolerance to innocuous allergens such as ovalbumin, thereby permitting allergen induced sensitization and airway disease. Our data might help to explain why active smoking is a risk factor for the onset of asthma [6].

## List of abbreviations

BAL: bronchoalveolar lavage

BALF: bronchoalveolar lavage fluid

CBA: cytometric bead array

CD: cluster of differentiation

CO: carboxyhemoglobin

DC: dendritic cell(s)

ELISA: enzyme-linked immunosorbent assay

ETS: environmental tobacco smoke

FACS: fluorescence activated cell sorter

GM-CSF: granulocyte-macrophage colony-stimulating factor

HBSS: Hank's balanced salt solution

IFN: interferon

Ig: immunoglobulin

IL: interleukin

OVA: ovalbumin

PAS: periodic acid-Schiff

Pbm: perimeter basement membrane

PBS: phosphate buffered saline

RL: lung resistance

SEM: standard error of the mean

TARC: thymus- and activation regulated cytokine

TCM: tissue culture medium

Th: T helper

TNF: tumor necrosis factor

## Competing interests

The author(s) declare that they have no competing interests.

## Authors' contributions

KM participated in the conception and design of the study, carried out the experiments and drafted the manuscript. LR contributed in the acquisition and analysis of data. TM has been involved in drafting the manuscript and revising it critically. GB participated in the critical reading of the manuscript. KT has been involved in the conception of the study design and in finalizing of the manuscript. GJ helped to conceive the study, and participated in its design and coordination and helped to draft the manuscript.
